# The ‘rejuvenating factor’ TIMP‐2 is detectable in human blood components for transfusion

**DOI:** 10.1111/vox.13023

**Published:** 2020-10-26

**Authors:** Julia Hoefer, Christian Dal‐Pont, Stefan Jochberger, Raffaella Fantin, Harald Schennach

**Affiliations:** ^1^ Department of Urology Medical University of Innsbruck Innsbruck Austria; ^2^ Central Institute for Blood Transfusion & Immunological Department University Hospital of Innsbruck Innsbruck Austria; ^3^ Department of Anesthesiology and Critical Care Medicine University Hospital of Innsbruck Innsbruck Austria

**Keywords:** blood components, blood transfusion, cognitive function, morphogenesis, tissue inhibitors of metalloproteinases

## Abstract

**Background and Objectives:**

Tissue inhibitor of metalloproteinases 2 (TIMP‐2) is a protein suspected to be crucial in numerous physiological and pathological processes such as morphogenesis, tissue remodelling and metastasis suppression. In animal models, the administration of TIMP‐2 to aged mice improved their cognitive functions. Therefore, one can hypothesize that differences in TIMP‐2 levels between blood donors and recipients might influence cognitive functions also in humans. However, the stability of TIMP‐2 during processing and storage of blood components for transfusion has not been intensively investigated so far. This study determined TIMP‐2 concentrations in fresh‐frozen plasma (FFP), erythrocyte concentrate (EC) and pathogen‐inactivated platelet concentrate (PI‐PC) depending on the donor's demographic factors age and gender.

**Materials and Methods:**

Tissue inhibitor of metalloproteinases 2 was measured in FFP (*n* = 30), EC (*n* = 12) and PI‐PC (*n* = 12) using a Q‐Plex single‐plex immunoassay for chemiluminescence‐based detection. Absolute quantification of TIMP‐2 was performed with Q‐view software. Fresh umbilical cord plasma was used as a positive control.

**Results:**

Tissue inhibitor of metalloproteinases 2 was detected in FFP (30/30 samples), EC (11/12 samples) and PI‐PC (12/12 samples). The median TIMP‐2 concentration in EC (17·2 ng/ml; range: 0–26·5 ng/ml) was significantly lower compared with FFP (63·4 ng/ml; range: 44·4–87·3 ng/ml) or PI‐PC (69·9 ng/ml; range: 39·9–83·6 ng/ml). Across all blood components, TIMP‐2 levels are comparable in male and female donors and independent of age.

**Conclusion:**

Tissue inhibitor of metalloproteinases 2 is detectable and stable in FFP, PI‐PC and, in low concentration, EC. It can be hypothesized that TIMP‐2 will be transmitted to recipients during transfusion.

## Introduction

In parabiosis models in which the blood streams of young mice were surgically connected to those of older animals, the young mice showed a decline in cognitive functions. This effect could be attributed to the chemokine eotaxin‐1 (CCL11), a molecule which has also been negatively correlated with memory function in human Alzheimer's disease patients [1, 2]. On the other hand, it has been demonstrated that human umbilical cord plasma revitalized hippocampal functions of aged mice [3]. The cognitive benefits conferred by cord plasma were mediated by tissue inhibitor of metalloproteinases 2 (TIMP‐2), a blood‐borne factor abundant in umbilical cord plasma as well as young mouse plasma and hippocampi. Taken together, these studies provide evidence of the presence of age‐dependent, blood‐borne factors which affect neurogenesis, cognitive functions and ageing when transferred to other individuals.

On the basis of these facts, it can be hypothesized that such ‘ageing’ or ‘rejuvenating’ factors are present also in blood components for transfusion and may be transferred to recipients during transfusion, which might affect the recipient`s cognitive capabilities. Indeed, it is known that a considerable proportion of patients experience a temporary or permanent decline in cognitive performance after major surgery, a phenomenon called postoperative cognitive dysfunction (POCD) [4]. The causal relationship of blood transfusion and such disturbances has not been clarified so far. However, intraoperative transfusion of erythrocyte concentrates (>3 units) has been described as an independent risk factor for the development of POCD [5]. Therefore, it is essential to investigate whether factors associated with cognitive function, neurogenesis and ageing are present in blood components processed and stored for transfusion.

In a previous study, we were able to demonstrate that the ‘ageing factor’ eotaxin‐1 is detectable and stable in ready‐to‐use blood products for transfusion. Most importantly, we discovered a gender‐independent increase of eotaxin‐1 with rising age of donors in both fresh‐frozen plasma (FFP) and erythrocyte concentrates (EC), while eotaxin‐1 was subject to only minor fluctuations within one donor over a longer period of time [6]. This is significant as high eotaxin‐1 levels are associated with decreased cognitive functions, although these were not examined in our previous study [1, 2, 6]. It might be possible that besides eotaxin‐1 also other factors associated with cognitive functions and ageing are detectable in human blood components processed for transfusion. TIMP‐2 has originally been described as an endogenous inhibitor of matrix‐metalloproteinases (MMPs). It is involved in MMP‐2 activation and contributes to the protection of the extracellular matrix (ECM) from proteolysis [7]. It has also been reported that TIMP‐2 is involved in neuronal differentiation and can act as a key effector of the pro‐neurogenic response to an inducing stimulus [8, 9]. Moreover, treatment of aged mice with either umbilical cord plasma or purified TIMP‐2 led to significant improvements in multiple cognitive measures such as contextual memory, learning and nesting, as well as increased synaptic plasticity in the hippocampus [3]. In addition, TIMP‐2 knockout mice display motoric dysfunctions such as dyskinesia, trembling and a splayed, shortened gait [10]. This phenotype has mechanistically been linked to altered formation and maintenance of neuromuscular junctions (NMJ), the deterioration of which is a common sign of ageing [11]. Taken together, these observations suggest that TIMP‐2 influences cognitive as well as motoric functions, which are both reduced in ageing. It is therefore possible that TIMP‐2 might affect cognitive capabilities and/or motor functions in recipients if transferred during blood transfusion.

As stated above, it has been shown that TIMP‐2 levels differ between young and old mice [3]. In humans, Rossignol et al. demonstrated age‐dependent changes. It has also been demonstrated that TIMP‐2 is present in freshly drawn human plasma [12, 13]. However, until now, it has neither been assessed if TIMP‐2 is detectable in human blood components processed and stored for transfusion, nor if TIMP‐2 correlates with age or gender in adult human beings.

In the present study, we assessed for the first time if blood products for transfusion (FFP, EC, PI‐PC) contain detectable TIMP‐2 levels and if TIMP‐2 concentration varies in relation to the donor`s age or gender.

## Material and methods

This trial was performed as a descriptive, single‐centre, non‐clinical pilot study. All experiments have been conducted in accordance with the Helsinki Declaration. The study was approved by the Ethics Committee of the Medical University of Innsbruck (Ethical vote number: 1083/2018).

### Investigational product

For the present study, we analysed leukocyte‐depleted blood products (FFP [*n* = 30], EC [*n* = 12], and PI‐PC [*n* = 12]) from volunteer donors aged 18 to 63. At the University Hospital of Innsbruck, the suitability for volunteer blood donation is assessed on the basis of stringent, standardized health criteria. After completing a medical questionnaire on their general health status, recent diseases, medication and travelling, all persons are physically examined, including measurements of blood pressure, heart rate, temperature and haemoglobin concentration. The donated blood is further tested for neopterin as well as transfusion‐transmissible infections such as HIV, hepatitis, syphilis and parvovirus B19. In case any disease or abnormality is detected, the donated blood is discarded. This procedure is to ensure all donors are in good health at the time of blood donation.

After donation, the blood components used for this study were stored under standard conditions at the Central Institute for Blood Transfusion & Immunological Department at the University Hospital of Innsbruck, Austria, after having undergone the following procedures:

### Preparation of erythrocyte concentrate

Leukocyte‐depleted ECs were prepared from single‐donor whole blood donations by cell separation. The preparation was conducted in accordance with the guidelines of the Council of Europe. One hundred millilitres of additive solution (SAGM) were added in order to increase shelf life. Each 280 ml EC package had a haematocrit of 50–70 vol% and contained less than 1*10^6^ leukocytes. ECs are stored for a maximum of 42 days at 4°C.

### Preparation of fresh‐frozen plasma

Leukocyte‐depleted FFPs were prepared from single‐donor whole blood donation by cell separation. FFPs at a core temperature of −30°C can be stored up to 2 years.

### Preparation of pathogen‐inactivated PC (PI‐PC)

Leukocyte‐depleted PCs were prepared by single‐donor apheresis. Each 300 ml PI‐PC contained 2–4*10^11^ platelets, suspended in approximately 35% plasma and 65% SSP + platelet additive solution. For safety reasons, pathogen inactivation treatment of all PCs was performed with amotosalen/UVA treatment (INTERCEPT blood system, CERUS corporation, Amersfoort, the Netherlands). PI‐PCs were stored under continuous agitation in gas‐permeable sterile plastic bags at room temperature (20–24°C). Under these conditions, the maximum storage time is 7 days.

### Sampling for the study

Aliquots from FFP (*n* = 30), EC (*n* = 12) and PI‐PC (*n* = 12) were taken from the respective blood component using a sterile connecting device.

In detail, samples used for the present study were prepared as follows:

All FFP samples (2 ml) were drawn at the end of the manufacturing process before freezing within 24 h after donation. Samples were then aliquoted (3 x 600 µl) and stored at −20°C until subjected to measurement 2–6 weeks later. EC samples (2 ml) were taken from the ready‐to‐use product within 24 hours after donation and stored at 4°C until subjected to measurement on the next day. PI‐PC samples (2 mL) were taken from the ready‐to‐use product within 24 h after donation and stored at 4°C until subjected to measurement on the next day.

All measurements of the respective blood product (FFP, EC, PI‐PC) were performed on the same day.

### TIMP‐2 measurement

A Q‐Plex single‐plex immunoassay for chemiluminescence‐based detection of human TIMP‐2 was employed according to the manufacturer's protocol (Quansys Biosciences, Logan, UT; range: 27·43 to 20 000 pg/ml; lower limit of detection: 15·9 pg/ml; intra‐assay coefficient of variation: 8%; inter‐assay coefficient of variation: 12%). The kit contained a freeze‐dried calibrator for solubilization in sample buffer and for generating a seven‐point standard curve. Signals were detected using ChemiDoc chemiluminescence imaging system (Biorad), and TIMP‐2 was quantified with Q‐View Software (Quansys) in relation to an 8‐point standard curve obtained by serial dilution of recombinant TIMP‐2. All samples were diluted 1:20 with sample dilution buffer and measured in duplicates. Plasma from fresh residual umbilical cord blood (*n* = 3, 2 ml each) was used as a positive control.

### Statistical methods

Statistical analyses were performed in SPSS and GraphPad Prism. Differences between groups were analysed using Mann–Whitney test. All differences highlighted by asterisks were statistically significant as encoded in figures (*P < 0·05, **P < 0·01, ***P < 0·001). Data are presented as Box–Whiskers Plots showing median and minimum to maximum values. For comparison of TIMP‐2 levels in relation to age, donors were divided into two groups, representing the younger and the older 50% each.

## Results

### Tissue inhibitor of metalloproteinases 2 is detectable in blood components for transfusion

**Fig. 1:**
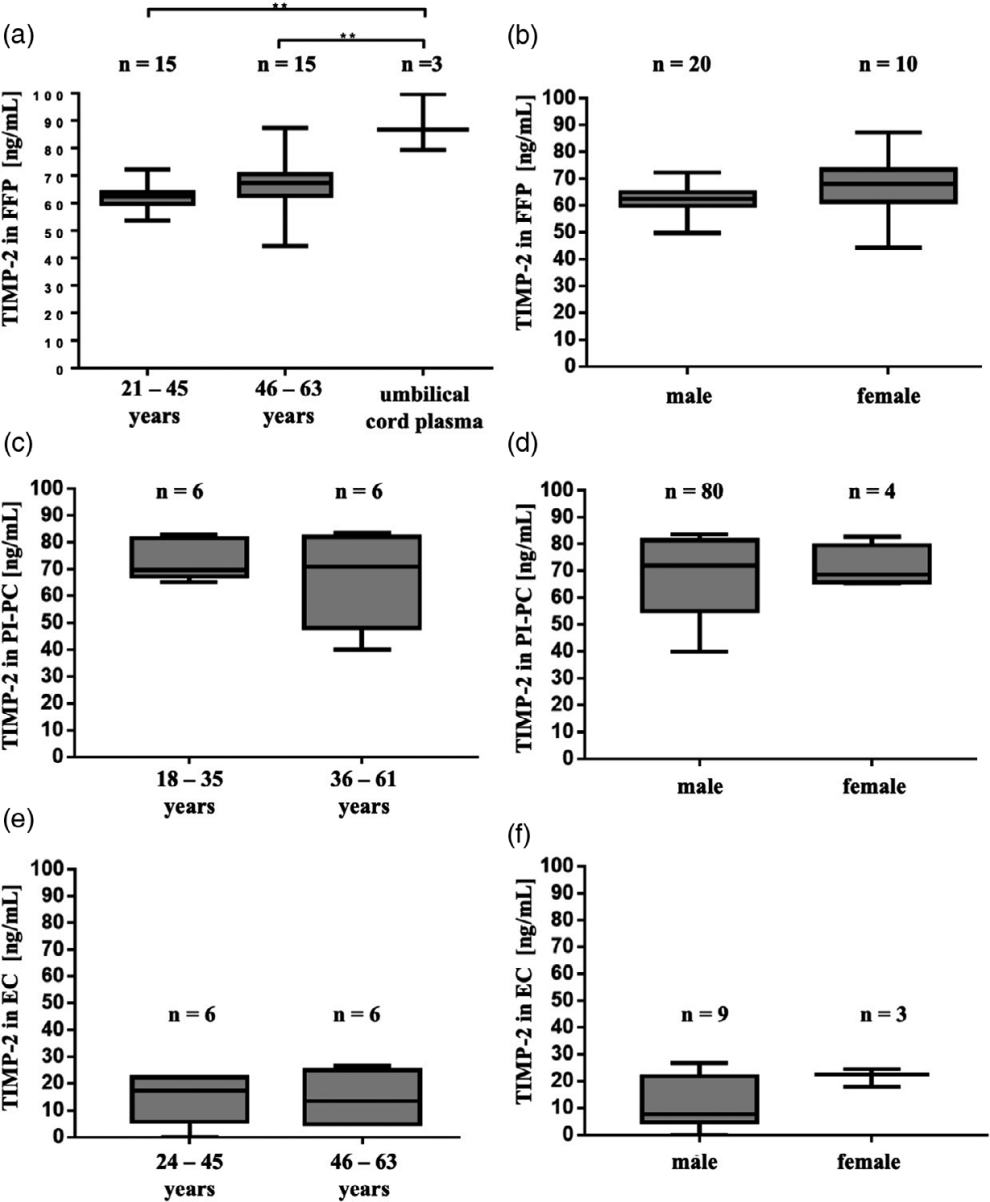
TIMP‐2 is detectable in fresh‐frozen plasma (FFP), pathogen‐inactivated platelet concentrate (PI‐PC) and erythrocyte concentrate (EC) (a,c,d). Umbilical cord plasma serves as a positive control. TIMP‐2 in FFP is comparable in male and female donors and independent of age (a,b). TIMP‐2 in PI‐PC is comparable in male and female donors and independent of age (c,d). TIMP‐2 in EC is rather low concentrated but comparable in male and female donors and independent of age (e,f). Data are presented as Box–Whiskers Plot, showing median and minimum to maximum values. *P < 0·05; **P < 0·01; ***P < 0·001; Mann–Whitney test.

Tissue inhibitor of metalloproteinases 2 was consistently detected in FFP (30/30 samples), PI‐PC (12/12 samples) as well as in EC (11/12 samples). Median TIMP‐2 levels were 63·4 ng/ml in FFP (range: 44·4–87·3), 69·6 ng/ml in PI‐PC (range: 39·9–83·6) and 17·2 ng/ml in EC (range: 0–26·5), as summarized in Table 1. Statistical analysis revealed significantly higher TIMP‐2 concentrations in FFP and PI‐PC, when compared to EC (Fig. 1).

**Table 1:** Summary of TIMP‐2 measurement

TIMP‐2 (ng/ml)	FFP (*n* = 30)	PI‐PC (*n* = 12)	EC (*n* = 12)
Minimum	44·4	39·9	0
25% percentile	60·08	65·65	4·98
Median	63·4	69·6	17·2
75% percentile	68·65	81·38	22·35
Maximum	87·3	83·6	26·5

FFP, fresh‐frozen plasma; EC erythrocyte concentrate; PI‐PC, pathogen‐inactivated platelet concentrate; TIMP‐2 values are presented as ng/ml.

### Tissue inhibitor of metalloproteinases 2 levels are not dependent on donor's age or gender

Regarding the question whether TIMP‐2 levels depend on demographic factors like age or gender, no significant differences in TIMP‐2 in FFP donated by younger (21–45 years) or older (46–63 years) individuals could be detected. However, umbilical cord plasma exhibited significantly higher TIMP‐2 levels compared with plasma from adult donors (Fig. 1a). Moreover, TIMP‐2 levels in FFP from male and female donors were comparable (Fig. 1b). Similarly, the median TIMP‐2 concentration did not differ in PI‐PC from younger (18–35 years) and older (36–61 years) donors (Fig. 1c), nor in PI‐PC samples from male or female individuals (Fig. 1f), although variability was higher in the older age group and in male donors. As mentioned above, the median TIMP‐2 concentration in EC was significantly lower compared with FFP or PI‐PC, and again, TIMP‐2 levels were comparable in EC samples from younger (24–45 years) and older (46–63 years) individuals (Fig. 1e), as well as in samples from male and female donors (Fig. 1f).

### Tissue inhibitor of metalloproteinases 2 is stable in FFP during storage

**Fig. 2:**
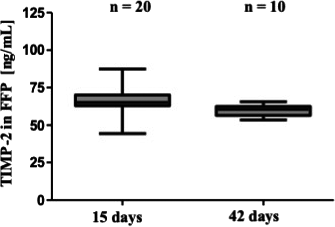
The Box–Whiskers Plot shows median and minimum to maximum values of TIMP‐2 level of FFP samples, which were stored for 15 and 42 days. No major differences in TIMP‐2 levels were detected.

Tissue inhibitor of metalloproteinases 2 was detectable in FFP after 15 days and 42 days of storage at −20°C. As shown in Fig. 2, no major differences in TIMP‐2 levels were found. Median TIMP‐2 levels were 65 ng/mL (range: 44·4–87·3) after 15 days of storage and 60·6 ng/mL (range: 53·5–65·6) after 42 days of storage (Table 2).

**Table 2:** TIMP‐2 is stable during storage

TIMP‐2 (ng/ml)	15 days (n = 20)	42 days (n = 10)
Minimum	44·4	53·5
25% percentile	62·95	56·48
Median	65·0	60·6
75% percentile	70·08	62·43
Maximum	87·3	65·6

TIMP‐2 is detectable in FFP (fresh‐frozen plasma) after 15 days and 42 days of storage at −20°C. TIMP‐2 values are presented as ng/ml.

## Discussion

Blood transfusion is a common medical procedure used for patients after serious injuries, certain diseases, or during major surgeries. EC, FFP and PI‐PC are routinely transfused without matching recipients and donors for gender, age and other demographic factors. It is therefore important to investigate the presence and stability of crucial blood‐borne factors in blood products. Moreover, it is essential to assess differences in the concentration of such factors in blood products from individuals with different demographic characteristics as well as the implication of these differences for the patient`s outcome after blood transfusion. TIMP‐2 is an interesting candidate for such an analysis, given that TIMPs are involved in numerous biological processes that require tissue remodelling and are thus key players in a range of physiological and pathological processes such as angiogenesis, wound healing, inflammation and cancer [14]. In addition, TIMPs influence hippocampal functions, cognitive measures and neurite outgrowth, and high TIMP‐2 in human umbilical cord plasma revitalized hippocampal functions of aged mice [2, 3, 14, 15, 16]. A previous study demonstrated that TIMP‐1 is detectable in platelet‐containing blood preparations [17]. However, the presence of TIMP‐2 in blood components has not been assessed so far.

In this descriptive pilot study, we demonstrated that FFP and PI‐PC contain comparable amounts of TIMP‐2 as reported in plasma concentrations of humans. On the other hand, TIMP‐2 was low to undetectable in EC. It is known that platelets contain MMPs and their inhibitors – among them TIMP‐2 – and that these factors are released into the plasma [18]. However, so far it has not been clear if TIMP‐2 is stable during the process of PI‐PC and FFP preparation and storage. Our data clearly show that TIMP‐2 is detectable in PI‐PC and FFP and thus will be transferred to recipients during transfusion. In our study, FFP exhibits a median TIMP‐2 concentration of 63 ng/ml, ranging from 44 to 87 ng/ml. Notably, in freshly drawn human plasma, the TIMP‐2 concentration has been found to range from 30 to 200 ng/ml, with a median TIMP‐2 concentration of approximately 80 ng/ml in healthy subjects [12, 13]. Furthermore, TIMP‐2 was detectable in similar plasma levels after storage of 15 and 42 days at −20°C. Thus, it can be concluded that TIMP‐2 is a relatively stable molecule that is not significantly affected during processing and long‐term storage of FFP.

We were also able to detect TIMP‐2 in EC, although levels were low compared with FFP and PI‐PC. Given that EC contains approximately 4% of residual plasma, in contrast to 35% of residual plasma in PI‐PC, a part of the detected amount of TIMP‐2 might derive from this source.

Interestingly, our data show that TIMP‐2 levels in all blood components are comparable across male and female donors and that TIMP‐2 does not correlate with the donor`s age. This is surprising, given that increased TIMP‐2 levels were found in young mice compared with older ones [3]. Concerning the dependence of TIMP‐2 levels in human plasma on age, trials showed discordant results. Tayebjee et al. found a modest negative correlation with age in healthy humans, whereas Bonnema et al. describe an increase of plasma TIMP‐2 levels with increasing age [19, 20]. However, more data are needed for clarification.

However, it has to be kept in mind that all blood products analysed in this study were of adult donors, with a minimum age of 18 years. Given that we and others found TIMP‐2 to be enriched in human umbilical cord plasma, it is possible that TIMP‐2 accumulates only during embryogenesis and the earliest years of childhood [3]. Nevertheless, we detected considerable differences in TIMP‐2 between individuals, especially in FFP. The reasons for these differences could not be assessed in this study. Processes that require tissue remodelling or wound healing might increase levels of MMPs and TIMPs; however, as mentioned above, one can hypothesize that all donors were in good health at the time of donation. Given the revitalizing effects of TIMP‐2 on the cognitive function of aged mice, it can be expected that a transfusion of FFP or PI‐PC containing high levels of TIMP‐2 to recipients with low TIMP‐2 might influence their cognitive measures as well [3]. However, this hypothesis has to be investigated in subsequent studies. Moreover, also other processes might be influenced by differences in TIMP‐2 in donor and recipient blood. For example, it has been shown in mice that MMP‐2 amplifies the response of platelets to weak stimuli, thereby promoting arterial thrombosis and that this process can be abolished by infusion of TIMP‐2 [21].

Taken together, our data clearly provide evidence that TIMP‐2 is present in FFP and PI‐PC, but neglectable in EC processed for transfusion and that TIMP‐2 levels vary between individuals. However, we did not detect any significant correlation with age or gender of the donors, although it has to be stated that the cohort of donors in this pilot study was small. Subsequent studies have to confirm these findings in a large population and further decipher the source of TIMP‐2 variability between individuals.

## Funding

This work has been supported by the Austrian Science Fund (FWF) grant T 738‐BBL, the Hertha Firnberg fellowship programme to JH and the Austrian Red Cross Dr. Karl Landsteiner Funding. The funders had no role in study design, data collection and analysis, decision to publish or preparation of the manuscript.

## Conflict of Interests

All authors declare no conflict of interest.
